# Improving Dissolution and Cytotoxicity by Forming Multidrug Crystals

**DOI:** 10.3390/molecules25061343

**Published:** 2020-03-16

**Authors:** Xufei Bian, Lan Jiang, Jing Zhou, Xiaoshu Guan, Jingyu Wang, Peng Xiang, Junyi Pan, Xiangnan Hu

**Affiliations:** 1 Department of Medicinal Chemistry, School of Pharmacy, Chongqing Medical University, Chongqing 400016, China; bianxufei95@163.com (X.B.); zhoujing045@cqmu.edu.cn (J.Z.); 17082343045@163.com (X.G.); 13340228643@163.com (J.W.); 13320227532@163.com (P.X.); 13399841645@163.com (J.P.); 2 College of Environment and Resources, Chongqing Technology and Business University, Chongqing 400016, China; jianglanlucky@163.com

**Keywords:** Rosiglitazone, Metformin, multidrug crystal, crystal structure, dissolution rate, cytotoxic effect

## Abstract

Both rosiglitazone and metformin have effects on blood glucose regulation and the proliferation of liver cancer cells. Combination therapy with these two drugs is common and effective for the treatment of diabetes in the clinic, however, the application of these two drugs is influenced by the poor dissolution of rosiglitazone and the gastrointestinal side-effect of metformin resulting from a high solubility. The formation of a multidrug crystal form (Rsg-Met) by a solvent evaporation method can solve the solubility issue. Crystal structure data and intramolecular hydrogen bonds were detected by X-ray diffraction and infrared spectroscopy. Surprisingly, Rsg-Met shortens the time spent in solubility equilibrium and multiplies the dissolution rate of Rsg. Finally, we found that a low concentration of Rsg-Met enhanced the proliferation inhibition effect on liver cancer cells (HepG2, SK-hep1) compared with rosiglitazone, without affecting the human normal cell line LO2.

## 1. Introduction

Among drugs for type 2 diabetes mellitus (T2DM) used in its clinical treatment, rosiglitazone (Rsg) and metformin (Met) are the first line agents [1]. Rsg, a representative drug of the thiazolidinedione family, control blood sugar by increasing insulin sensitivity [2]. Conversely, Met can inhibit gluconeogenesis in livers [3,4,5] but has no effects on insulin secretion. Thus, the combination of these two drugs is commonly used to improve the effect of T2DM treatment because of their complementary mechanisms [6].

Recently, extensive research has shown that Rsg and Met can decrease the occurrence rate of some cancers and improve cancer prognosis in T2DM patients, especially for liver cancer [7,8,9,10,11,12]. The primary reason may be that both glycemic control and tumor inhibition are closely linked to energy and metabolism and the liver plays a vital role in regulating energy and metabolism [13,14,15,16,17,18,19]. Therefore, we anticipated that the combination of Rsg and Met would have a cytotoxic effect on liver cancer cells via a complementary effect.

However, on the basis of the Biopharmaceutical Classification System (BCS), Rsg is ranked as a class II molecule with low solubility and high permeability [20]. It is now well established that bad dissolution can impair the processes of absorption, distribution, metabolism and excretion (ADME). Thus, the performance of Rsg is limited by its poor dissolution. Conversely, Met is ranked as a class III molecule due to its high solubility and low permeability [21]. Met can therefore release rapidly after taking it and directly stimulate the local gastrointestinal mucosa because of its excessive solubility [22]. The high occurrence rate of this adverse effect limits its application in clinical treatment. Generally, the optimal processing method was applied to handle this issue [23,24,25]. These strategies can be effective but are expensive. 

In this context, medicine modification through crystal engineering and supramolecular techniques offers a route. We could synthesize a multidrug crystal aimed at T2DM patients with liver cancer. Co-crystal synthesis is a relatively simple approach to modify an existing active pharmaceutical ingredient (API) to achieve desirable properties, such as improved dissolution [26]. Multidrug crystals generally incorporate an API with a pharmaceutically-acceptable molecule in their crystal lattice [27,28,29]. In the preparation processes, the co-crystals could generate novel hydrogen bonds without any structural modification of the parent drug [30]. Therefore, the novel multidrug crystal can retain or enhance the original pharmacological activities of the API.

**Figure 1:**
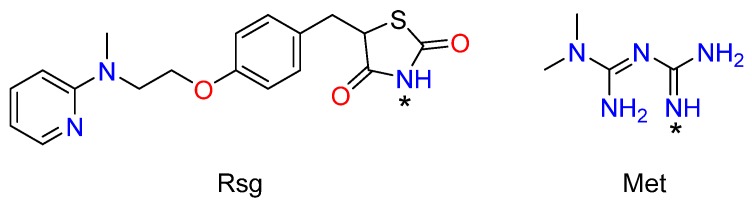
Chemical structures of the Rsg and Met with basic and acidic sites highlighted (*), respectively.

Additionally, from the structural point of view, Rsg and Met possess multiple sites which have potential hydrogen bonding capacities (Figure 1). The guanidine group of Met is one of the best hydrogen-bonding acceptors [31]. Both the lactam group of Rsg and the amine group of Met also have the ability to form a variety of hydrogen-bonding motifs in crystals, so it is feasible to obtain a novel multidrug crystal consisting of Rsg and Met.

In this article, we aimed to synthesize a novel multicomponent crystal which we have named Rsg-Met. We also performed structural analysis and dissolution rate of this multidrug crystal. Finally, its cytotoxicity effect was detected by the MTT assay, which indicated that Rsg-Met had a better inhibitory effect on some liver cancer cell lines (HepG2, SK-hep1) compared with the mixture and Rsg alone.

## 2. Results

### 2.1. The Characterization of Crystal Structure

**Figure 2:**
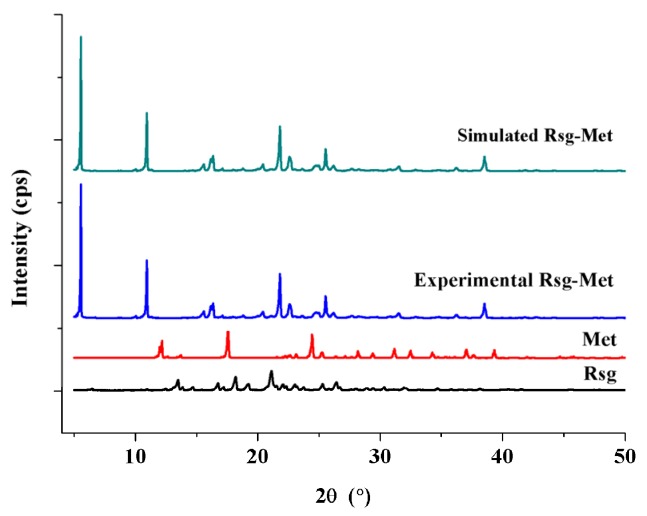
X-ray powder diffractograms of Rsg-Met, Met and Rsg.

Powder x-ray diffraction (PXRD) was used for the preliminary detection. Clearly, the patterns of Rsg-Met (5.4, 10.5, 16.2, 22.3, 23.6, 26.8, 38.1) are different from those of the Met (13.2, 18.1, 24.9, 32.0, 34.7, 37.6) and Rsg (15.2, 15.6, 17.4, 18.2, 20.1, 22.4, 32.2), as shown in Figure 2. 

**Figure 3:**
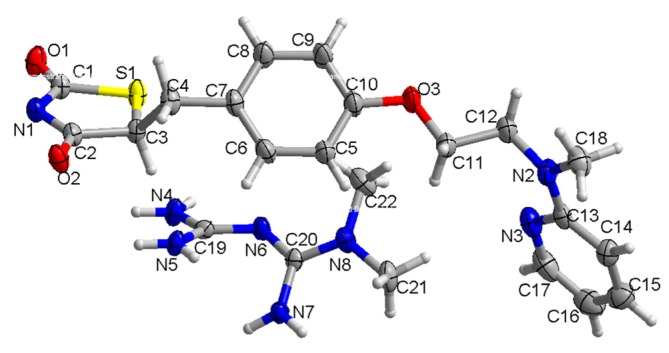
Thermal ellipsoid figure for the Met ion and Rsg ion molecules drawn at 50% probability level.

Following the disappearance of the characteristic peaks of two pure ingredients, the appearance of novel characteristic peaks in the Rsg-Met spectra was recorded. Moreover, the experimental PXRD patterns of Rsg-Met are similar to the simulated patterns of the corresponding single crystal. The similarity with the simulated powder pattern shows that the prepared Rsg-Met powder is a pure phase. The crystallographic data of Rsg-Met, listed in Table S1 (CCDC: 1962489) was collected by single crystal X-ray diffraction (SCXRD). There were one molecule of Rsg and one molecule of the Met in the asymmetric unit (Figure 3).

**Figure 4:**
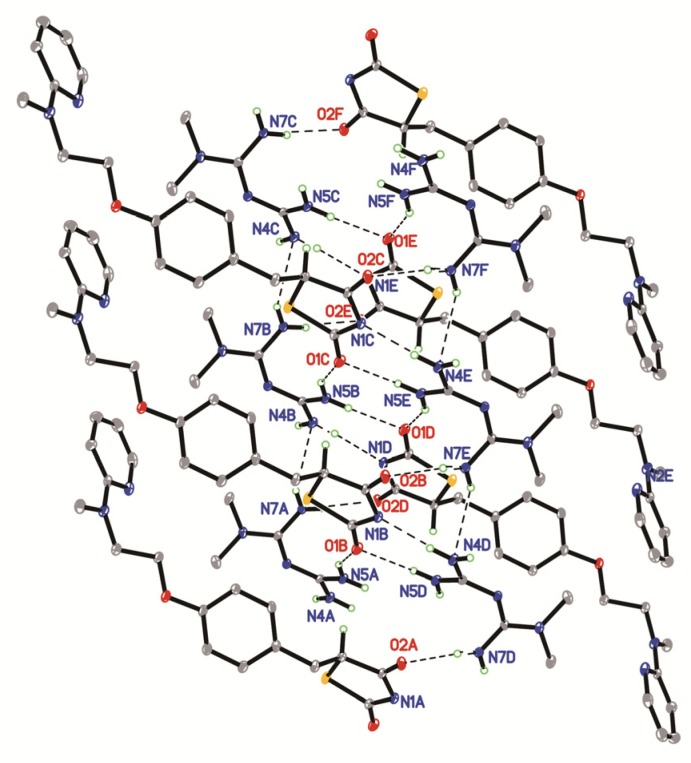
Two dimensional layered structure connected by hydrogen bonds of Rsg-Met.

Rsg-Met is characterized by complex 2D hydrogen-bonded layers parallel to the (1 0 0) plane, as illustrated in Figure 4 and Table 1. To be specific, Met contributed to a strong hydrogen bonding by the two guanidine groups connecting with the lactam group and the thiolactone group of the Rsg molecule, respectively (N(5)-H(5A)...O(1), N(7)-H(7A)...O(2)). These two molecules also linked via the amine-amide supramolecular effect (N(4)-H(4D)...N(1),). Interestingly, this network was observed to constitute the whole crystal unit. It was linked with the N-H···N (N(4)-H(4C)...N(6), N(7)-H(7B)...N(4)) interactions between Met molecules. 

The most surprising aspect of the data is the molecules of Rsg and Met were connected not only by hydrogen bonds, but also by an ionic bond (Figure 1). More specifically, the H atom at the nitrogen labelled N1 between the lactam group and the thiolactone group of Rsg moved to the imine group of Met (Figure 3). Thus, the stability of the intermolecular structure was maintained by hydrogen bonds, ionic bonds, and van der Waals forces.

**Figure 5:**
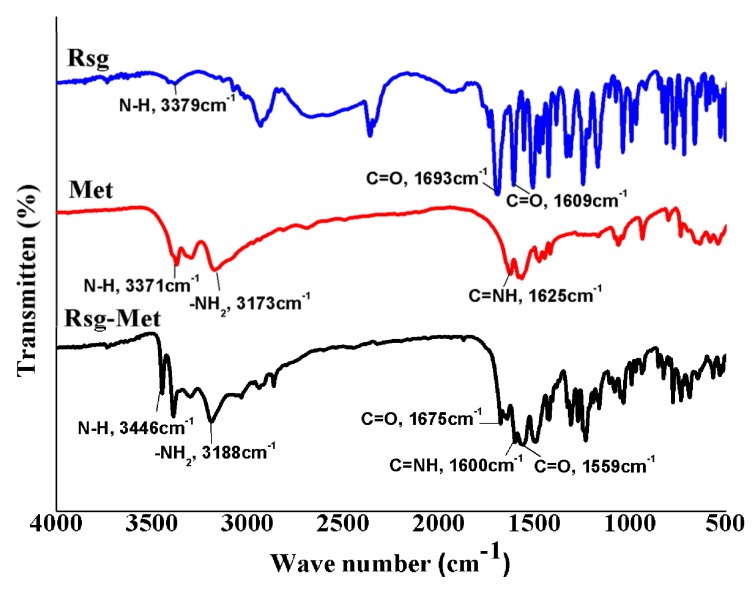
The FT-IR spectrum of Rsg, Met, and Rsg-Met.

Fourier-transform infrared (FT-IR) spectroscopic analysis is a standard detection method. In the first instance, the Rsg-Met IR spectrum should be a juxtaposition of the IR spectra of the single ingredients. There were slight shifts in absorption as the corresponding groups involved in strong hydrogen bonding and ionic bonding. This indeed was the case, as shown in Figure 5.

In the infrared spectra of the Rsg-Met, the N-H vibration (3379 cm^−1^) of Rsg moved to high-frequency and showed a novel absorption peak at 3446 cm^−1^, which was influenced by the H atom of N between lactam and the thiolactone from Rsg moving to a biguanide bond from the Met molecules. The thiolactone C=O vibration (1693 cm^−1^) and the lactam C=O vibration (1609 cm^−1^) of Rsg moved to low-frequency and showed a novel absorption peak at 1675 cm^−1^ and 1559 cm^−1^, which were influenced by producing a slight electron pair effect due to the formation of N anions. Additionally, the N-H vibration (3371 cm^−1^), -NH_2_ vibration (3173 cm^−1^) and C=NH vibration (1625 cm^−1^) from Met moved to high/low-frequency compared with the corresponding groups in the pure Met material, respectively. Overall, these results indicate that Rsg-Met formed hydrogen bonds and ionic bonds between Rsg and Met.

### 2.2. The Enhancement of the Dissolution Rate

**Figure 6:**
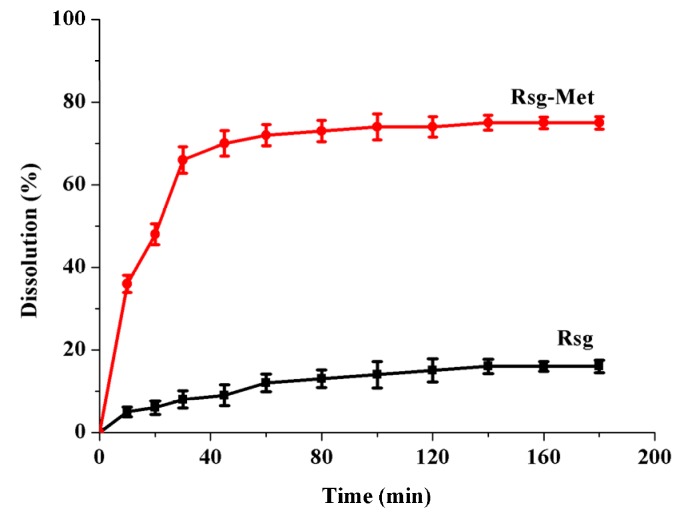
Dissolution rate of Rsg and Rsg -Met in alkaline medium (pH = 6.8).

It is necessary to enhance the poor dissolution of Rsg due to its classification in the BCS. Additionally, since Rsg is a weak base, the absorption of Rsg in the gastrointestinal tract occurs predominantly in the intestine (pH = 5–7). Therefore, we conducted this experiment in phosphate buffer (pH = 6.8). As can be seen from the Figure 6 below, the Rsg-Met group reported significantly and consistently higher dissolution than Rsg alone during the whole period. The dissolution rate of Rsg-Met reached equilibrium at the 60 min point. At the end, it peaked at a highest point of around 75%.

Notably, Met powder was directly soluble when it contact with an alkaline medium (pH = 6.8) [21,22], so it was impossible to acquire any dissolution rate data, hence there are no Met results in this test and we concluded that the Met was an extremely soluble compound. In this context, the trend depicting the dissolution rates of these samples was as follows: Met > Rsg-Met > Rsg.

### 2.3. The Cytotoxicity on Liver Cancer Cells

**Figure 7:**
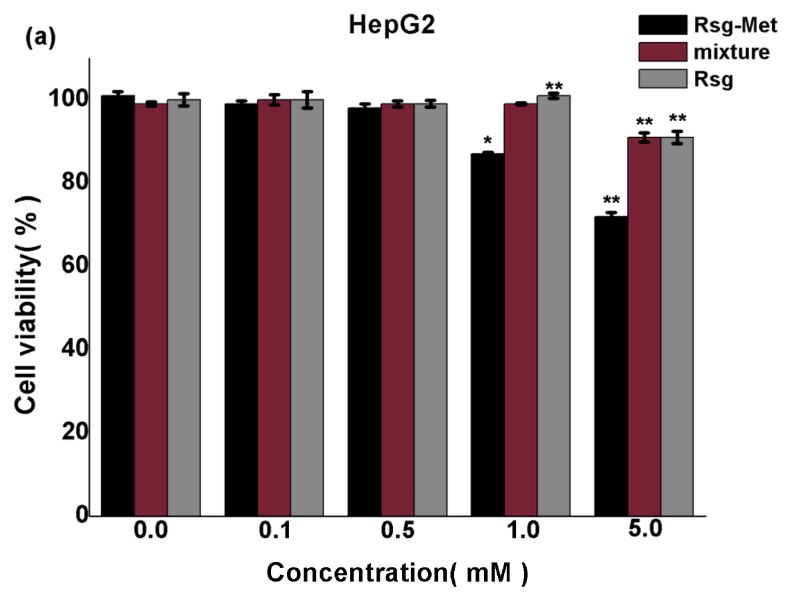

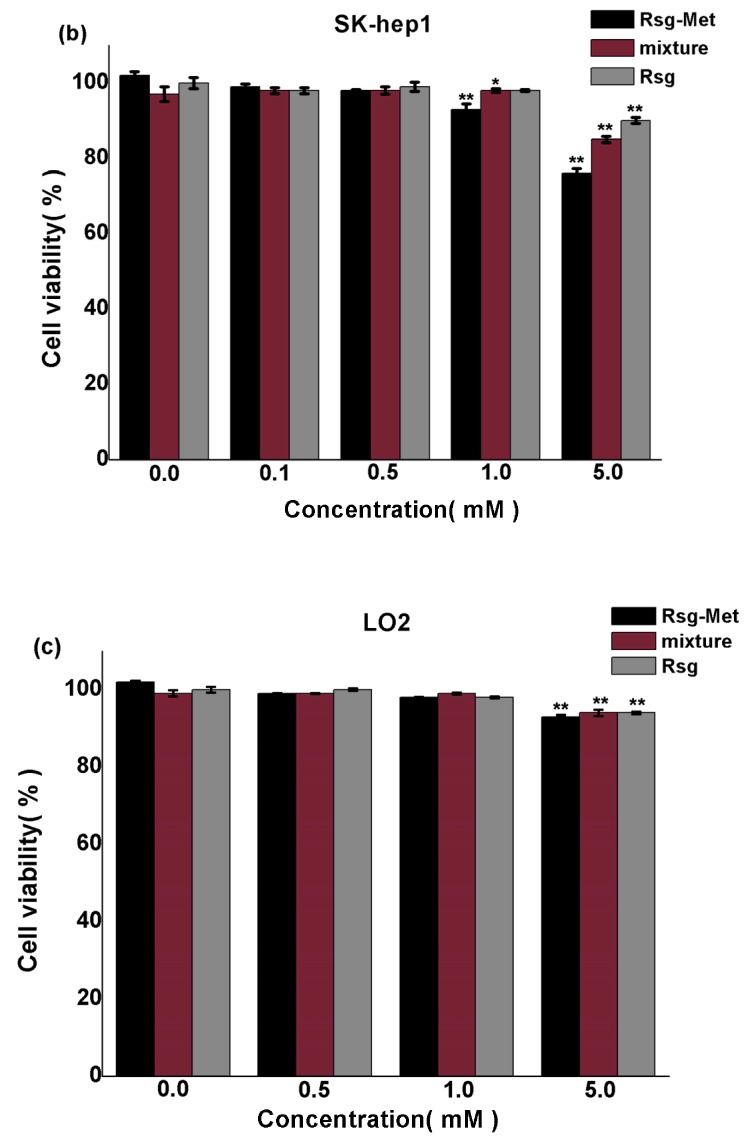
Detection of drug toxicity to (**a**) HepG2, (**b**) SK-hep1, and (**c**) LO2 cells by MTT assay treated with Rsg-Met, the mixture of Rsg and Met at the molar ratio of 1:1 and Rsg (* *P* < 0.05 and ** *P* < 0.01 versus the control group).

The results of the MTT assay are shown in Figure 7 and the cytotoxicity ranking was the Rsg-Met > Rsg and Met mixture > Rsg. It was found that Rsg-Met inhibited proliferation of two human hepatoma cell lines (HepG2 and SK-hep1) in a dose-dependent manner. The mixture and Rsg also caused a dose-dependent inhibition in liver cancer cells, however, a significant reduction of proliferation was found in the presence of the Rsg-Met at 1 mM, but not with the other two agents (Figure 7a,b). It was suggested that Rsg-Met was not acting as a simple sum of the two pure components but rather as a novel compound which had higher efficacy at the same concentration than the components individually. Plus, no significant difference was observed when LO2 normal human liver cell line cells were treated with low doses of Rsg-Met (Figure 7c), so it was concluded that a low concentration of Rsg-Met enhanced the anti-proliferative effect on liver cancer cells without affecting the human normal cell line LO2.

## 3. Discussion

Rsg and Met are classical oral anti-diabetic drugs used for T2DM patients [32]. The combination of the two agents is common in the clinic to improve the hypoglycemic effect [33]. The most common formulation is 500 mg Met and 4 mg Rsg and the molar ratio of Met and Rsg is 275:1. [34]. However, Met has a high incidence rate of gastrointestinal reactions and the main reason is its high solubility [35]. Conversely, Rsg has poor solubility [20]. Therefore, we hoped to solve the solubility issue by preparing a multidrug crystal with an appropriate molar ratio of Met and Rsg.

Additionally, recent epidemiologic studies have suggested that both Rsg and Met have an anti-hepatoma effect [11,36]. As the liver is a critical organ in regulating energy and metabolism, the mechanisms of glycemic control and cancer inhibition share some features [37,38]. Therefore, the combination of Rsg and Met may have better liver cancer cytotoxic performance.

In this context, Rsg-Met was created with the basis of supermolecular chemistry and crystal engineering. A simple and cheap process was applied to prepare this novel multidrug crystal. The single most striking observation to emerge from the data analysis was the enhancement of the dissolution and cytotoxicity of Rsg.

The SCXRD data showed that the two pure ingredients were integrated by novel intramolecular hydrogen-bonds (N-H...O, N-H...N) and ionic-bonds. One molecule of Rsg-Met consisted of one molecule of Rsg and one of Met. From the perspective of hydrogen bonding analysis, Rsg-Met reduces the opportunity to form hydrogen bonds between Met and H_2_O molecules because most of the hydrogen bond donor and acceptor moieties in Met actually formed hydrogen bonds between Met and Rsg molecules. Correspondingly, with the introduction of the highly soluble Met molecule, the dissolution of Rsg-Met was enhanced.

Furthermore, we found that Rsg-Met had higher efficacy in inhibiting the proliferation of HepG2 and SK-hep1 liver cancer cells at lower concentrations than the mixture and Rsg and it has no effect of the human normal cell line LO2. This may be as the result of the improved dissolution of Rsg while a complementary effect between Rsg and Met cannot be excluded [39].

The results of the experiments enhance our knowledge about these classical hypoglycemic agents, especially for Rsg. They provide an alternative method to address the problems related to poor solubility and the traditional fixed high-dose combinations. It also offers a potential application in the design and development of relevant and similar clinical anti-liver cancer drugs for T2MD patients.

## 4. Materials and Methods

### 4.1. Materials

Rsg was purchased from Adamas Reagent Company (Shanghai, China) and used as received. Met was prepared by adding metformin hydrochloride (0.65 g, 0.004 mol) and sodium hydroxide (0.1 g, 0.004 mol) into 70 mL of ethanol and filtering the suspension after stirring at 25 °C for 12 h, followed by solvent removal with a rotary evaporator. The obtained free base of Met was freshly used in the next experiments. Other chemicals were purchased from Adamas Reagent Company, and used without any further purification.

### 4.2. Solvent Evaporation Method

Boiling ethanol (35 mL) containing Rsg (1.43 g, 0.004 mol) was added to the same volume of ethanol containing Met (0.52 g, 0.004 mol) and vigorously stirred. The resulting solution was covered by parafilm perforated with a few small holes, and allowed to evaporate slowly under a surrounding temperature of 20 °C. After three days, this process yields colorless triclinic-shaped crystals that are suitable for single crystal x-ray diffraction. The obtained solids were filtered and dried under room conditions for further characterization.

### 4.3. Powder X-ray Diffraction (PXRD)

PXRD data for the crystalline products were collected using a Bruker D8 Advance x-ray diffractometer (Bruker, Karlsruhe, Germany), operating in transmission geometry with Cu Kα radiation (λ = 1.5406 Å), 40 kV/100 mA, and fitted with a LynxEye linear detector (Bruker, Karlsruhe, Germany). The samples were prepared on silicon single crystal sample holders with a 20 mm depth. Data for each sample were collected from 2θ = 5° to 50° at 25 °C with step and scan speed of 5 °/min.

### 4.4. Single Crystal X-ray Diffraction(SCXRD)

SCXRD data were collected on a SMART CCD diffractometer (Bruker, Karlsruhe, Germany) using Cu-Kα radiation (λ = 1.54184 Å) with a graphite monochromator at 293 K. The integrated and scaled data were empirically corrected for absorption effects with spherical harmonics, implemented with the SCALE3 ABSPACK scaling algorithm. Using Olex2 [40], the structure was solved by the ShelXS [41] structure solution program using direct methods and refined with the ShelXL [42] refinement package using least squares minimization. All non-hydrogen atoms were refined with anisotropic displacement parameters. The hydrogen atoms of N guanidine and N amide were located from the differential Fourier map and were refined with isotropic displacement parameters. All other hydrogen atoms were obtained geometrically.

### 4.5. Fourier-Transform Infrared (FT-IR)

Mortars and pestles were previously washed and placed in a dryer for 30 min. Then, each compound (1 mg) was ground into powder with dried KBr (50 mg) in a mortar. The mixture was pressed into a piece of slice and recorded on a Spectrum FT-IR spectrometer (Thermo Scientific Nicolet, Waltham, MA, USA) in the range of 4000–400 cm^−1^.

### 4.6. Dissolution Rate

The dissolution rate of Rsg and Rsg-Met in powder form was studied by using a US Pharmacopoeia tablet dissolution test apparatus (paddle method, Hanson Research, Chatsworth, CA, USA) in 900 mL of phosphate buffer (pH 6.8) containing 0.25% (*w*/*v*) of sodium lauryl sulfate as a dissolution medium under constant temperature 37 °C and 75 rpm. The powder equivalent to 100 mg of Rsg was weighed and added into dissolution medium. Ten mL samples were withdrawn at 10, 20, 30, 45, 60, 80, 100, 120, 160, 180 min, and fresh medium added at the same volume and temperature at the same time to maintain the volume constant. Three mL of the secondary filtrate was taken by filtering with 0.45 μm filter (Titan, Shanghai, China) and discarding the primary filtrate. The UV-Visible detector (UV2600, Shimadzu, Kyoto, Japan) was set at a wavelength of 318 nm. Every procedure describe above was performed three times.

The Lambert-Beer law was used to measure concentration of samples, and the equation is C=El/A. In this formula, A means absorbance measured by UV-Visible detection, E is a constant, l means thickness of absorption layer which generally is 1 cm, and C means concentration. The data were detected by UV-Visible detection and linear regression were performed in the concentration (C, μg/mL) by peak area (A) that equation was: A = 0.2244 + 0.098C (R^2^ = 0.9995), which valid from 50 to 90 μg/mL. Then, the dissolution rate can be measured by the equation w = CV/m × 100%. In this formula, w means the dissolution rate, C means concentration, V means 10 mL which were withdrawn from samples and m means 100 mg Rsg.

### 4.7. MTT Assay

The human hepatoma cell lines HepG2, SK-hep1 and immortalized normal human liver cell line (LO2) were obtained from the Chinese Academy of Sciences Cell Bank (Shanghai, China). Cells were cultured in DMEM (Gibco, Carlsbad, CA, USA) supplemented with 10% fetal bovine serum (FBS) and 1% penicillin/streptomycin under 37 °C and 5% CO_2_ humidified condition. Logarithmically growing cells were treated with indicated concentrations of each compound for 48 h and then replaced with regular culture medium.

The cellular cytotoxic effect was evaluated using Cell Titer96^®^ Aqueous One Solution Cell Assay Kit (Promega, Madison, WI, USA). 2 × 10^3^ cells were plated into each well of 96-well plates and incubated in regular culture medium for 6 h. Then, cells were treated with indicated concentrations (0.1 mM, 0.5 mM, 1.0 mM and 5.0 mM) of drugs for 48 h. 20 μL MTT reagents were added into each well and incubated at 37 °C for 2 h. The absorbance was measured at 490 nm by enzyme-linked immunosorbent meter. Experiments were performed in triplicate.

The data from individual experiments were presented as the mean ± SD. Statistical comparisons between groups were done using one-way ANOVA followed by Dunnett post hoc testing. *p <* 0.05 was considered statistically significant using SPSS 22.0.

## Figures and Tables

**Table 1:** Hydrogen bonds for Rsg-Met.

D-H...A	d(D-H)/Å	d(H...A)/Å	d(D...A)/Å	D-H-A/°
N(4)-H(4C)...N(6) ^1^	0.847	2.263	3.110	178
N(4)-H(4D)...N(1) ^2^	0.88	2.00	2.720	138
N(5)-H(5A)...O(1) ^3^	0.84	2.14	2.988	178
N(5)-H(5B)...O(1) ^2^	0.91	2.19	3.083	167
N(7)-H(7A)...O(2) ^4^	0.86	2.26	3.086	161
N(7)-H(7B)...N(4) ^3^	0.93	1.89	2.758	154

Symmetry transformations used to generate equivalent atoms: ^1^ 1 − x, −y,1 − z; ^2^ −x, 1 − y,1 − z; ^3^ 1 + x, +y, +z; ^4^ 1 − x,1 − y,1 − z.

## Supplementary Materials

The following are available online at https://www.mdpi.com/1420-3049/25/6/1343/s1, Figure S1: The FT-IR spectrum of Rsg, Figure S2: The FT-IR spectrum of Met, Figure S3: The FT-IR spectrum of Rsg-Met, Table S1: Crystal data and structure refinement for Rsg-Met. The crystallographic information file (cif) of this study is deposited at the Cambridge Crystallography Data Center with deposit number 1962489. This data can be obtained free of charge from The Cambridge Crystallographic Data Centre via www.ccdc.cam.ac.uk/data_request/cif.
