# Supplementary material for: Improving Dissolution and Cytotoxicity by Forming Multidrug Crystals
Source: Molecules. 2020 Mar 16;25(6):1343. doi: 10.3390/molecules25061343 (PMC7144552; doi:10.3390/molecules25061343)
Supplement: Supplementary file 1 [file molecules-25-01343-s001.pdf]

The supplemental files

Table S1. Crystal data and structure refinement for Rsg-Met.

Empirical formula	$C_{22}H_{30}N_8O_3S$
Formula weight	486.60
Crystal system	triclinic
Space group	P-1
a/Å	6.9482
b/Å	11.3147
c/Å	11.3147
α/°	72.375
β/°	82.370
γ/°	74.153
Volume/Å^3	1240.82
Z	2
D_{calc} g/cm^3	1.302
μ/mm^{-1}	1.493
2θ range for data collection/°	8.46 to 134.146
Reflections collected	16699
Independent reflections	4433 [R_{int} = 0.0390, R_{sigma} = 0.0305]
Data/restraints/parameters	4433/18/334
Goodness-of-fit on F^2	1.026
Final R indexes [I> 2σ (I)]	R_1 = 0.0523, wR_2 = 0.1428
Final R indexes [all data]	R_1 = 0.0629, wR_2 = 0.1550
Largest diff. peak/hole/e Å^{-3}	0.43/–0.28
CCDC no.	1962489

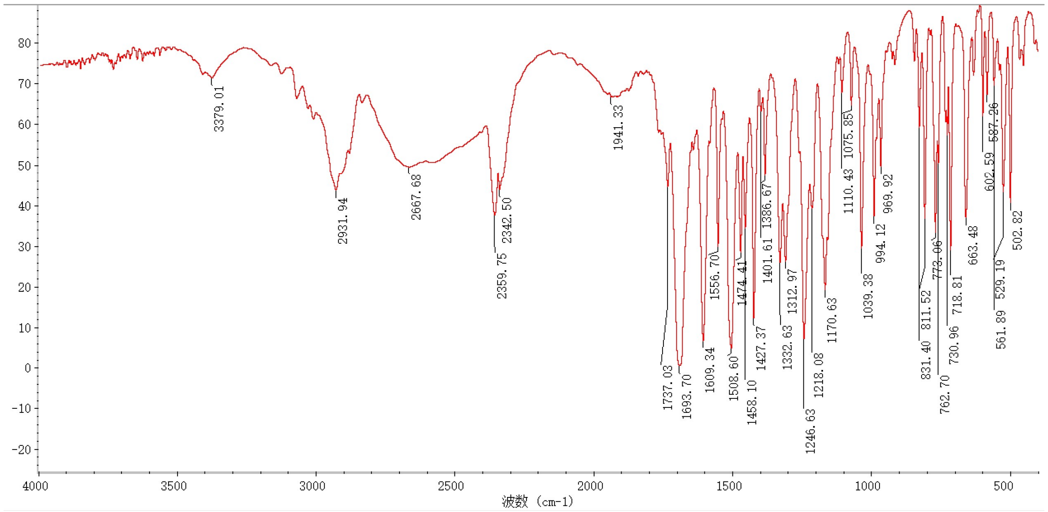

Figure S1. The FT-IR spectrum of Rsg

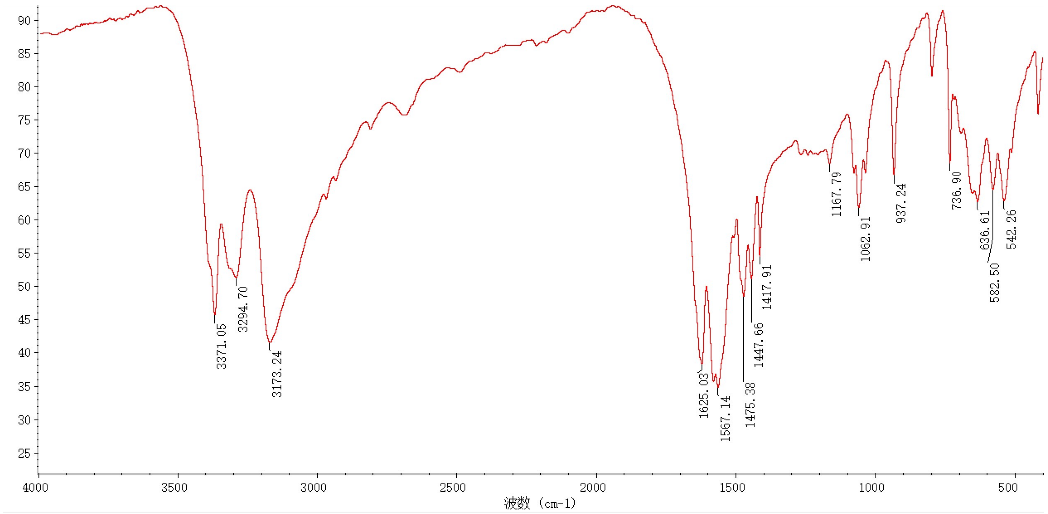

Figure S2. The FT-IR spectrum of Met

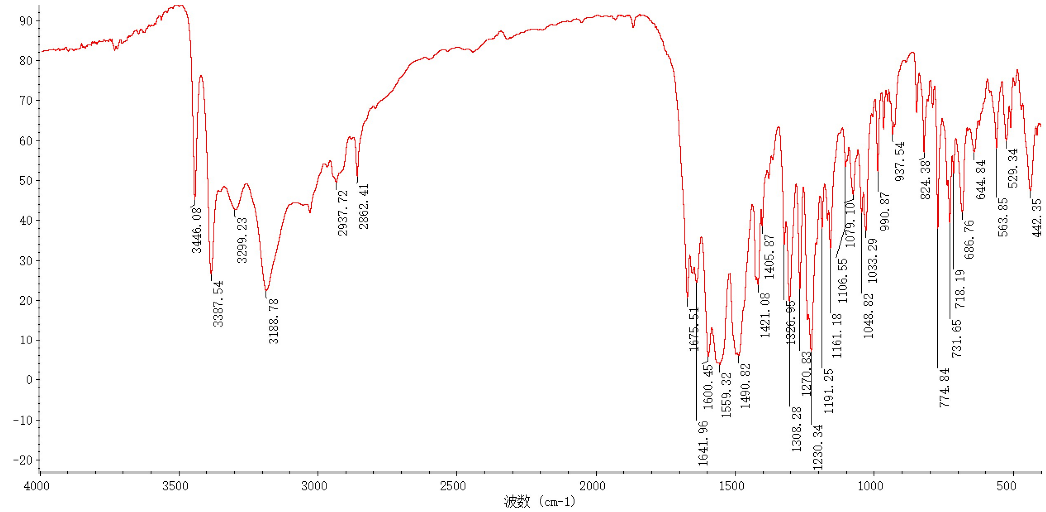

Figure S3. The FT-IR spectrum of Rsg-Met
